# ‘The big buzz’: a qualitative study of how safe care is perceived, understood and improved in general practice

**DOI:** 10.1186/s12875-018-0772-z

**Published:** 2018-06-09

**Authors:** Carl de Wet, Paul Bowie, Catherine O’Donnell

**Affiliations:** 1 Medical Directorate, NHS Education for Scotland, Glasgow, UK; 2 General Practice & Primary Care, Institute of Health & Wellbeing, College of Medical, Veterinary and Life Science, University of Glasgow, Glasgow, Scotland; 3 School of Medicine, Griffith University, Southport, Gold Coast, Australia

**Keywords:** Patient safety, Patient safety incidents, General practice, Family medicine, Quality improvement

## Abstract

**Background:**

Exploring frontline staff perceptions of patient safety is important, because they largely determine how improvement interventions are understood and implemented. However, research evidence in this area is very limited. This study therefore: explores participants’ understanding of patient safety as a concept; describes the factors thought to contribute to patient safety incidents (PSIs); and identifies existing improvement actions and potential opportunities for future interventions to help mitigate risks.

**Methods:**

A total of 34 semi-structured interviews were conducted with 11 general practitioners, 12 practice nurses and 11 practice managers in the West of Scotland. The data were thematically analysed.

**Results:**

Patient safety was considered an important and integral part of routine practice. Participants perceived a proportion of PSIs as being inevitable and therefore not preventable. However, there was consensus that most factors contributing to PSIs are amenable to improvement efforts and acknolwedgement that the potential exists for further enhancements in care procedures and systems. Most were aware of, or already using, a wide range of safety improvement tools for this purpose. While the vast majority was able to identify specific, safety-critical areas requiring further action, this was counter-balanced by the reality that additional resources were a decisive requirment.

**Conclusion:**

The perceptions of participants in this study are comparable with the international patient safety literature: frontline staff and clinicians are aware of and potentially able to address a wide range of safety threats. However, they require additional resources and support to do so.

## Background

In the last few decades, a growing body of evidence suggests that patient safety incidents (PSIs) commonly occur and that a substantial minority result in preventable, iatrogenic harm to patients in primary care [1–3]. Improving care quality and safety is now a priority in many modern health care systems, including the UK National Health Service (NHS). Consequently, a diverse range of improvement initiatives and interventions have been developed and implemented, ranging from small-scale, informal actions in single units, practices or care teams to formal collaborative-type large-scale programmes at regional and national levels [4–6].

The Scottish Patient Safety Program (SPSP) is an example of this approach [7]. It initially focused on specific, high-risk processes in secondary and tertiary care centres only, before being extended into primary care [4]. As a result, there are now a range of potential improvement methods, tools and interventions available that have been adapted and contextualised for the general practice setting [8–15].

Despite these initiatives and a growing research agenda, there is still limited evidence that the standards of care, including in general practice, have been significantly improved as a result of specific interventions. While there are many potential reasons for this, they typically relate to two interlinked issues. The first is the characteristics of the intervention, in particular whether it is useful, acceptable, feasible and implemented as intended by those who developed and tested it. The second issue is whether the intended users of these complex health care interventions are willing and able to effectively implement them and continue to use them.

In order to fully understand these issues, it is therefore important, and possibly essential, that health care policy makers and researchers elicit and attempt to understand the perceptions of frontline staff groups (the ‘on-the-ground’ implementers) before attempting to implement such complex interventions, not least because these perceptions largely determine how an intervention is understood and implemented in routine practice [16]. However, to date, the perceptions of general practice team members remains, for the most part, unknown [17].

## Method

### Study aims

The aims of this study were threefold:

1. To describe the perceptions, understanding and experiences of safe care from a range of general practice team members in Scotland;
2. To identify and describe the issues perceived as important contributing factors to patient safety incidents (PSI); and.
3. To identify existing improvement actions and strategies and explore team perceptions about potential opportunities for future interventions.

### Design

This is a qualitative study, using semi-structured interviews, conducted with general practitioners (GP), practice nurses (PN) and practice managers (PM). The decision to interview different team members was motivated by the multidisciplinary nature of general practice, its strong ethos of integrated working to explore clinical and non-clinical perceptions and experiences of how safe care is understood and delivered.

### Setting and sample

The study was undertaken in Scotland in two mainland NHS Health Boards: one covering a large, urban setting with 262 general practices (designated Health Board A); the other covering a mixed urban-rural setting, with 56 practices (Health Board B). For the purposes of this study practies were considered ‘semirural’ if they were in outlying areas adjacent to suburbs. In April 2012, all practice managers in each Board area were sent written information via e-mail about the proposed study and an invitation for the PM, one GP and a PN to participate. Due to time and resource constraints, a convenience sample of the first 12 GP practices who responded was constructed: 10 practices from Board A and 2 from Board B.

### Data collection

An open-ended interview guide was developed based on a scan of the international patient safety and implementation science literature and from previous experience of the authors. A single, one-to-one interview was conducted in the practice premises of each participant at a time convenient to them, starting in July 2012 until June 2013. As part of the sampling process, the interviews occurred in a fluid manner between the different practices and team members. Prior to the interviews, the reasons for the research were explained to the participants and informed consent was obtained. They were assured that the research team genuinely wanted to understand their perceptions of patient safety and that this would help inform potential improvement initiatives. All interviews were conducted by the same investigator (CdW), who introduced himself as a GP and researcher. Interviews were digitally recorded and supplemented with contemporaneous fieldnotes.

### Data analysis

All interviews were transcribed verbatim but were not reviewed by participants. Transcripts were anonymised and the twelve participating practices assigned a unique, double digit identifier. A thematic analysis was undertaken, which allowed the identification of emergent codes and themes. The six stages described by Braun and Clarke were followed, namely: familiarisation with data; generation of initial codes; searching for themes among codes; reviewing themes; defining and naming themes; and producing the final report [18]. The codes and themes were mapped and displayed with NVivo version 9.2.81.0. The number and nature of the themes are described in the results section.

The analysis aimed to be emergent and exploratory and was not intended to identify numeric differences in responses. The data were independently coded by CdW and COD and all authors met regularly to discuss the findings, ensure consistency and agree and verify data interpretations. There were also regular meetings with a research peer who were not directly involved in the study and who did not have a background in direct clinical care. This was considered valuable as these offered perspectives that were not shaped by personal experience of general medical practice.

## Results

The demographic data of the participating practices are summarized in Table 1. A total of 34 interviews were held with 11 general practitioners, 12 practice nurses and 11 practice managers. One participant had the dual role of practice nurse and manager and one GP initially agreed to participate but had to withdraw due to personal reasons. Although the final sample size for the study was fixed a priori, analysis eventually stopped identifying new themes and content, indicating that theoretical saturation was achieved.

**Table 1:** Demographic data of the participating practices

Practice no	Patient list size^a^	GPs (n)		Area	Training practice (Yes/No)
		Partners	Other		
1	2100	1	–	Semi-rural	No
2	4300	3	1 salaried	Urban	Yes
3	3200	1	1 salaried	Urban	No
			1 long-term locum		
4	4100	3	1 Retainer	Urban	Yes
5	11,000	8	–	Semi-rural	Yes
6	5900	4	1 Salaried	Urban	Yes
7	8200	7	–	Urban	Yes
8	6800	3	2 Salaried	Urban	Yes
9	6400	3	1 Salaried	Urban	No
10	9900	6	1 Retainer	Urban	Yes
11	3000	4	1 Retainer	Urban	Yes
12	7500	6	1 Salaried	Urban	Yes

^a^At the time of the interviews, rounded to the nearest hundred

### Perceptions and experiences of ‘patient safety’

A few participants were able or attempted to provide a ‘working’ definition of patient safety, but most felt that a formal definition of this notion had little value for them. However, all were able to articulate what patient safety meant to them in practical terms.

*It’s about taking personal and professional responsibility for that patient... not passing the buck, taking responsibility for each patient (PN02)*

The themes that emerged were that patient safety is: (i) *important*; (ii) *integral* to care; (iii) characterized by *impermanence*; and (iv) amenable to *improvement efforts*.

#### Patient safety is important

All participants agreed that patient safety is important; some considered it *the* most important characteristic of care. However, with other equally important priorities competing for limited time and resources, the relative importance of patient safety fluctuated over time.

*[Patient safety] is almost the ‘be all and end all’. Whatever you do you have to make sure that patient safety is your highest priority (GP11)*

*It’s just I suppose for most practices, it’s just something else to do in your already crammed up day (PM10)*

Circumstances that increased its importance within the practice were after detection and reporting of a significant PSI or as a result of the practice team participating in an improvement programme promoted by the Health Boards.

#### Patient safety is integral to care

The vast majority of participants felt that patient safety had been an integral part of their practice for many years, even if they had not explicitly acknowledged this. According to them, external agencies had only recently started to show an interest in this area with the result that it was becoming *‘fashionable’*, *‘sexy’* (GP04) and a *‘buzz word’*. As an example, some of the participants referred to the SPSP that had been launched nationally in 2008 and to local enhanced services being promoted by their NHS Health Boards to illustrate national and regional policy interest in this area.

*We’ve been doing this same thing under different names for years. It has been going on for years. It’s just been called different things (PM04)*

#### Patient safety is characterized by impermanence

The majority of participants understood patient safety as the dynamic and emergent product of many different and variable processes in health care, all in temporal relationships with each other. Many explained the impermanence of safety by referring to patient journeys within health care. They described patient journeys as unpredictable, including a wide range of health care staff with the potential to influence the ‘destination’, e.g. whether PSIs occurred or safe care was delivered.

*It’s a whole kind of journey, and we’re involved in so many aspects of it. There’s safety in the physical viewing, there’s safety in the medical assessment, medical administration and then the ongoing care, and I suppose secondary care. You know, you can talk about the journey into it secondary care as well. (GP07)*

#### Patient safety is imperfect but can be improved

All but one of the participants thought PSIs were the inevitable consequence of complex clinical care provision and even if ‘infinite’ resources were theoretically available, they could never be completely prevented. Some therefore described the possibility of ‘perfect’ patient safety as a ‘pipedream’ or a ‘wish’. Despite this, all agreed that this was not an excuse for focusing on PSI prevention or reduction and that much could still be done in this regard. They also unanimously agreed that improving patient safety was an ethical and professional responsibility for everyone working for the NHS. However, according to them, the expectations about the results of these efforts should be tempered by the acknowledgement that clinicians are ‘human’ and therefore imperfect and prone to err.

*Total patient safety is [pause] will I say unachievable? I don’t think it’s ever going to be achievable, but I think there’s a lot of things can be done to change things, but I don’t think you’ll ever get 100% perfect - you’ve got too many ingredients for that, too many ingredients (PN08)*

### Factors perceived as important contributors to PSIs

Participants identified many different factors which they felt contributed to PSIs. These can be summarized in five main groups: (i) chance; (ii) inadequate time and resources to deal with increasing workloads; (iii) lack of care continuity; (iv) patient-related factors; and (v) clinician-related factors.

#### Chance

Most considered ‘chance’ or ‘luck’ to be the most important contributing factor to many PSIs and the resultant harm severity. Consequently, many respondents expressed feeling helpless and unable to prevent these types of PSIs in the future because, as one GP explained, *‘if she were to have that same day again, probably the same thing would happen again. It’s just a set of circumstances... you might be lucky, you might be unlucky’* (*GP02).* However, all participants acknowledged that chance was not always implicated, nor the main contributing factor for every PSIs.

#### Inadequate time and resources to deal with increasing workloads

All of the participants described how they, and the rest of their practice teams, were struggling to safely manage their existing workloads which continued to increase. They responded to the increasing workloads with a range of formal and informal adaptive behaviours, including changing appointment systems, working additional, unpaid hours and choosing to forego breaks and meals. Despite these behaviours, they were aware that potential safety threats still remained and in some instances had even increased. For example, participants reported the potential for inappropriate patient triage, prescriptions being signed without being reviewed and reception staff sometimes offering patients appointments with team members who were not clinically appropriate to their needs. Acceptable and feasible solutions were difficult to implement, as procuring additional time and staff directly reduced the income and livelihood of the partners and could, in some, instances make the practice non-viable.

*The pressure that is on practices to churn out patients and churn out facts, figures, returns - it’s phenomenal (PM02)*

*At my lunch break I’m putting information on the computer (PN03)*

#### Lack of care continuity

Continuity was generally perceived as an important contributing factor to safe care. Conversely, a lack of continuity was perceived to have a negative impact, particularly during care transitions between health care providers and at the interface between different organizations*.* Many participants were concerned that care continuity was being eroded at a practice level, which they attributed to increasing workloads, patient expectations of same-day consultations and the increasing reliance on locum staff.

*Sometimes we’ve had issues over the last few years and I think really it’s because people are darting in and out and don’t really know what’s going on (PM12)*

#### Patient-related factors

The majority of participants – and especially the practice managers - felt patient expectations and health care needs had increased to the point where it was difficult or impossible to meet these and this now posed a potential threat to effective and efficient care delivery. This feeling was reportedly compounded by some patients who were perceived as not taking at least some responsibility for their own health, patients with significant clinical complexity and the increasing prevalence of multimorbidity in ageing practice populations. The clinicians reported struggling, and often failing, to effectively and safely manage the *‘shopping lists’* (PN08) of patients in 10 min consultations. However, while some patients were perceived as demanding, participants acknowledged that the majority of patient requests and expectations were appropriate and that clinicians and practices were responsible for meeting them.

*I think they [patients] have some real unrealistic expectations of what doctors can and can’t do (PM03)*

#### Clinician-related factors

Participants described a wide range of factors relating to the behavior and attitudes of clinicians that may contribute to but also help to prevent PSIs. In some cases, clinicians were making unintentional assumptions about care, although it was recognized that *‘Assumptions aren’t good for safety’* (GP02). In other cases, clinicians were making intentional changes that were not part of system recommendations or procedures. Reasons for assumptions and violations included workload, ongoing distractions or competing priorities for time.

*The systems are there. People either don’t follow the systems or don’t have time so try to cut corners with the systems, and that’s why things fail (PM07)*

One of the more insidious factors that some participants reported contributed to PSIs was the issue of ‘personalities’ in their teams. Participants used the word ‘personality’ euphemistically to explain their observations that the specific characteristics of a few clinicians seemingly increased the risk of PSIs occurring in the practice. These characteristics included being ‘afraid to ask for help’; lacking insight about their own knowledge and skill deficiencies; and interpersonal relationships and communications skills that made it challenging for others to raise concerns about potential safety threats with them.

*[There is] huge variance from practice to practice and I guess it’s all about the GPs that you work with (PM08)*

*Nobody is perfect ever, so you have to be aware of your limitations (PN09)*

### Existing and potential future improvement actions

Participants described many potential methods and tools that they were aware of or had used to improve patient safety. These included formal and informal interventions and their scope ranged from small changes for a single patient to changing or creating new policies and procedures for the practice. Examples of the different types of improvement actions participants described are provided in Table 2 and illustrated with selected quotes.

**Table 2:** Examples of improvement methods and actions participants already use

Action or method	Selected verbatim quotes
*‘Formal’ actions*	
Significant event analysis (SEA)	We do significant events regularly... we will meet to discuss it (PM06)
Clinical audit	We do lots of audits around [access] and check that it’s still as good as we think it is, and we occasionally have to tweak the amount of triage (PM08)
Protocols	Over the last few years with being a training practice we have tried to put a lot of protocols and systems in place to protect it (PN05)
CPD, appraisal and revalidation	Individually you are doing the best for the patient that you have and that is your responsibility, so there is a bit about professional development, CPD and maintaining your knowledge and recognising your weaknesses (GP08)
*‘Formal’ and informal actions*	
Involving patients	We’re calling it ‘complaints, comments and compliments’ and what we’re asking, we’ll go out regularly and speak to the patients and say ‘how do you feel about how we’re doing? Is there anything we can improve on?’ How do we know we’re completely safe? I think this is maybe a way of us checking are we doing enough (PM02)
*Informal actions*	
Raising awareness of safety critical issues	People are making others aware of what has happened and that is the way forward and we will just continue to do that, and hopefully we will get better and better at it (PM06)
Sharing information / peer feedback	I think being able to discuss things with my nursing colleague - on a Wednesday I start at one, we have an hour’s handover - I find that really useful (PN02)
Mitigation, esp. pharmacists and patients	I think there are lots of sources that stop us from falling short more of the time, to be honest (GP03)

When asked to suggest high-priority patient safety areas for future intervention, the majority of participants identified two specific issues. The first issue was medication and medication-related processes. Participants were concerned about the very large volume of repeat prescriptions generated on a daily basis, usually by administrative team members, which were then typically signed without clinical review by GPs. They felt that more, and more thorough, medication reviews would be useful to help prevent PSIs. The second issue was ensuring housebound patients receive high-quality care, with some participants suggesting the creation of nursing roles specifically to care for this group of patients or incentivising practices to provide this service.

Despite recognizing potential areas for improvement, participants unanimously agreed that they had no time, resources or spare capacity to even consider implementing these suggestions, or *any* other new interventions. They also doubted whether any other general practice team could feasibly undertake any additional, unfunded work. Some participants also expressed concern that quality improvement interventions may increase workload and paradoxically decrease patient safety by reducing their time to provide clinical care. One respondent explained that this was why *‘it [patient safety initiative] can be seen as hassle for some’* because *‘it takes us out from the day to day practice when I have still got other patients and normal work carries on’ (GP06).*

## Discussion

The study sought to understand the views of general practice staff with respect to patient safety in routine practice. All agreed that patient safety is important and understood it to be an integral part of the care they provide. Participants identified many factors that they felt were important contributors to PSIs, many of which could potentially be detected and mitigated. However, most also felt that a proportion of PSIs are an inevitable consequence of complex care delivery, and therefore not amenable to any intervention. Despite this perception, they unanimously agreed that patient safety can and should be improved. Most participants were aware of and could describe a wide range of strategies that have been used in their practices to improve standards of care. They were also able to recommend specific ‘high risk’ areas which they felt should be prioritized for safety improvement interventions in the future. However, everyone strongly agreed this would only be feasible if additional resources were provided.

### Comparison with existing literature

The contributing factors to PSIs identified in this study are similar to those reported by clinicians in other health care settings or working in different countries. For example, a qualitative study in the USA explored the recollections of family physicians (*n* = 53) of their most memorable errors and the perceived causes through in-depth interviews. Similar to this study, many different possible contributing factors (*n* = 34) were considered, which the authors categorized as: physician stressors and characteristics; process-of-care factors; and patient-related factors [19]. Additionally, a survey of clinicians (*n* = 848) working in outpatient settings in the USA identified many cognitive and system factors considered to be related to diagnostic errors [20].

Inadequate resources, including lack of time, and increasing workloads were not only perceived as important safety threats by the study participants, but were also understood to have a negative impact on the performance and wellbeing of clinicians and staff. This finding is comparable with the international patient safety literature. In a focus-group study of primary care physicians (*n* = 32), lack of resources and time pressures were perceived as particularly important impediments to care quality, with the authors’ reporting that inadequate resources ‘*often force physicians to compromise standards of care’* [21]. Insufficient time to provide all of the necessary care patients require is a well-recognized and important contributing factor to PSIs. A framework identifying specific types of ‘time problems’ in general practice was recently proposed and include the ‘office tempo’, which describes and quantifies the amount of time clinicians have available to provide care for patients [22]. All the participants in this study identified this ‘tempo’ as a particularly important risk factor for PSIs.

The two ‘high-risk’ areas for PSIs identified as being particularly suitable for future interventions were medication-related processes and housebound patients. These perceptions are similar to those of GPs in the Netherlands who indicated in a web-based survey that prescribing and monitoring of medication and patient age over 75 years were the most important safety risks [23]. These issues have comprehensively been described in the international patient safety literature and are widely recognized as important areas for further research [24].

The prevailing perception in this study that some PSIs are inevitable and cannot be prevented may seem overly pessimistic or even fatalistic to some, depending on their understanding of patient safety. From a ‘psychological’ perspective PSIs can be explained as the linear cause-and-effect results of individual human error, which in turn can be attributed to finite physiological and psychological resources [25]. Accordingly, *all* health care workers are susceptible to err, the likelihood of error increases as the number of ‘demands’ on human resources increase, and the frequency and type of errors are largely predictable – and therefore manageable. However, this simplistic explanation fails to acknowledge that human error is a necessary and important mechanism through which we all learn.

An alternative perspective about the contributing factors to PSIs would be that they result from technical and systems failures. These failures can be represented by linear models in which it is possible to identify simple, complex and cascading causes, contributing factors and outcomes [26]. A PSI is explained as the product of a series of events which occur in a specific and (retrospectively) recognizable manner and therefore allows knowledge about the future. The implication is that some PSIs may be prevented by detecting and eliminating potential threats proactively and by designing, incorporating and strengthening health care system defences.

### Practical implications

The finding in this study that only a few participants were able to provide a formal definition of patient safety is comparable to the international literature. In a qualitative study of GPs (*n* = 22) and practice nurses (*n* = 7) in the Netherlands, none of the respondents provided a definition of patient safety, although they were able to offer a wide range of descriptions and perceptions of this concept when asked [27]. One implication of this finding is that the way in which researchers and policy makers define patient safety may not have any practical meaning for frontline care staff. In order for improvement interventions to be successfully implemented, it may therefore be necessary to first align the understanding of all stakeholders about patient safety.

The fact that all participants could provide practical examples of how they already use improvement tools and methods could be interpreted as evidence of their willingness to consider other, new interventions. On the other hand, there is a danger that any new intervention may be perceived as ‘just’ another tool in a toolbox that is already full. However, one of the key findings of this study is that for future interventions to be feasible, additional resources would first have to be provided.

### Limitations and strengths

Approximately a third of all Scottish general practices provide GP specialty training for registrars [28], while the majority of practices in this study had training status. This was because of our sampling strategy, which was a pragmatic choice based on the resources available and access to general practices through their association with NHS Education for Scotland. The perceptions of the sample may therefore not be representative of all general practices in Scotland or other countries in the UK. On the other hand, practices were selected from two NHS Health Boards and included training and non-training, small (single practitioner) and larger practices from urban and semi-rural geographical locations.

By its nature, interview data confines analysis to what people report or their perceptions associated with the phenomenon under inquiry. However, interviews were candid, detailed and in-depth, all participants had considerable professional autonomy and the interviews were conducted by a clinical peer. It is therefore unlikely that they offered socially desirable responses. The reflexivity and rigour of the research was increased through data clinics to refine the coding framework. The perceptions and experiences of three different staff groups were explored in order to reflect the multidisciplinary reality of modern general practice. The perceptions of the vast majority of participations were highly congruent despite their different roles. While the sample size was determined beforehand, thematic saturation was achieved and more interviews would not have materially strengthened the main findings.

## Conclusion

The perceptions of participants in this study are comparable with the international patient safety literature, namely that patient safety is important, integral to the delivery of care and that there are potential opportunities for improvements in general practice systems and procedures. However, any further improvements at the level of individual general practices in the UK are contingent on investment of additional resources. The study findings also suggest a need for a more integrative approach to patient safety improvement efforts at a national level, i.e. incorporating components from human factors, systems and resilience engineering.
